# The disconnect in hepatitis screening: participation rates, awareness of infection status, and treatment-seeking behavior

**DOI:** 10.7189/jogh.09.010426

**Published:** 2019-06

**Authors:** Cheryl Lin, Rachel Clark, Pikuei Tu, Rungting Tu, Ya-Jung Hsu, Hsiao-Ching Nien

**Affiliations:** 1 Policy and Organizational Management Program, Duke University, Durham, North Caroloina, USA; 2 College of Management, Shen-Zhen University, Shenzhen, China; 3 Liver Disease Prevention and Treatment Research Foundation, Taipei, Taiwan; 4 Graduate Institute of Clinical Medicine, College of Medicine, National Taiwan University, Taipei, Taiwan; * Joint first authors

## Abstract

**Background:**

Over 325 million people in the world are infected with hepatitis B or C virus. Chronic hepatitis is responsible for 78% of cases of hepatocellular carcinoma and an estimated 1.3 million preventable deaths a year. As “silent killers”, liver diseases are often asymptomatic and go undiagnosed until their terminal stage. Knowledge of infection status via screening is thus a vital part of preventing spread and seeking early treatment. Recently there has been a worldwide push to eliminate hepatitis. The objective of this study is to assess hepatitis B and C self-reported awareness of infection status vs correct awareness (compared to blood test results) and follow-up rates in Taiwan to inform global health promotion efforts that utilize screening interventions to prevent chronic liver diseases.

**Methods:**

De-identified data from a Liver Foundation’s nationwide community-outreach free blood screening programs was utilized, including 50 909 participants’ data from 74 sites with a questionnaire (demographics, screening history, hepatitis awareness, monitoring behavior) and blood test results. Chi square tests were applied using R programing to examine the impacts of demographic variables on infection prevalence, awareness, and behavior relating to hepatitis.

**Results:**

Among all participants, 41.1% indicated having had a hepatitis screening, of which only 60.8% knew their results. Around 69.7% and 66.5% self-reported awareness of their hepatitis B and C status respectively; 12.8% and 26.4% of individuals who tested positive for HBsAg and Anti-HCV respectively incorrectly thought they were not infected. Of those who self-reported awareness of their positive infection, 43.4% and 26.6% did not follow up with a health care professional for monitoring or treatment; the top reasons were “no symptoms”, “too busy”, and “don’t know where to follow up”. Rural populations showed higher infection prevalence but lower screening rates and self-reported awareness.

**Conclusions:**

Intervention programs must address the substantial number of people that do not recall if they were screened or do not know the results of a screening. Discrepancies between self-reported awareness, correct awareness, and follow-up and disparities across demographic groups deserve further scrutiny. Global hepatitis eradication initiatives should reconsider how screening, test results, and education are presented in order to improve awareness and prevent chronic infection that could develop into life-threatening liver diseases.

Over 325 million people in the world, the size of the United States population, are chronically infected with hepatitis B or C virus (HBV or HCV), the leading causes of liver cirrhosis and hepatocellular cancer [1,2]. Both HBV and HCV are communicable through blood and bodily fluids and therefore transmission is a serious public health concern [3-5]. Chronic hepatitis – defined as being infected for more than 6 months – is responsible for 57% of cases of liver cirrhosis, 78% of cases of hepatocellular carcinoma, and an estimated 1.3 million preventable deaths a year worldwide [2,6]. For HBV, the risk for chronic infection is related to age at infection: approximately 50% of infected children (ages 1 to 5 years) become chronically infected compared with 5% to 10% of adults [7,8]. In contrast, hepatitis C becomes chronic for 75% to 85% of those infected with HCV [7]. Because the liver has no nerve endings and can function with 70% of its mass damaged, liver diseases are commonly referred to as “silent killers”: They are often asymptomatic and go undiagnosed until their terminal stage [9,10]. Knowledge of hepatitis infection status is thus a vital step in preventing spread and seeking early treatment. Wide-scale blood screening is a key intervention tool used to inform people of their hepatitis status and subsequently direct and motivate those infected towards appropriate health behaviors and follow-up care [11]. Unfortunately, past studies have indicated insufficient screening availability, low participation rate, and suboptimal self-monitoring or adequate treatment of those who have been infected [12,13].

Studies have reported a wide range of population awareness rates for hepatitis infection. Shin et al’s study of chronic hepatitis patients in Korea revealed that 74.2% and 34.9% of people infected with HBV and HCV respectively were aware of their infection [13]. Another study focusing on HCV in the United States found that 49.7% of people were aware of their infection and 77.5% of those who tested positive had followed-up with a doctor [12]. A European Centre for Disease Prevention and Control (ECDC) report estimated the undiagnosed proportion of hepatitis cases in Europe ranged from 40% in Italy to 85% in Germany for HBV and from 20% in Denmark to 91.2% in Greece for HCV [14]. More alarmingly, the World Health Organization (WHO) predicted the awareness rates of people infected with chronic hepatitis were less than 10% in South-East Asia and 5% in Africa—two regions where the virus is most prevalent [15,16]. Furthermore, less than one in ten who know their hepatitis status in South-East Asia have access to treatment; in Africa, less than 1% in need of antiviral therapy receive care [15,16].

In light of a recent global effort to eradicate hepatitis [17,18], we set out to examine five critical and interrelated questions that largely determine the effectiveness of blood screening activities in helping prevent transmission, chronic viral infection, and the development of life-threatening liver diseases: (1) do people partake in screenings and, for those who do, know their test results; (2) are people aware of their hepatitis status; (3) is their awareness correct; (4) do people follow up and seek treatment once diagnosed; and (5) if not, what are the reasons. We also aimed to investigate demographic variations and possible disparities in these five questions in order to better inform future interventions targeting vulnerable populations. For this study, we define *awareness* as self-reported recognition of one’s own hepatitis infection status, and *in/correct awareness* as in/accurate self-reported knowledge of one’s infection status when verified against blood test results.

Hepatitis is exceedingly prevalent in Asia [19]; South Asia and East Asia account for 52% of the total number of hepatitis-related deaths in the world [20]. In Taiwan, cancer – of which liver cancer rates 2nd – is the leading cause of death, while cirrhosis and other chronic liver diseases rank tenth [21]. The government and private sector have made concerted efforts to eliminate hepatitis: Taiwan was the first country in the world to launch a national HBV vaccination program for all newborns in 1984 [22]. In 1995, the country established the National Health Insurance (NHI), a single-payer system which now covers more than 99% of the population with comprehensive, high-quality, and affordable care that has garnered above an 80% satisfaction rate for the program [23]. The NHI Administration introduced more comprehensive coverage for HBV and HCV treatments in 2003 with a sliding scale co-pay of US$0 to US$18 per doctor visit [24]. After a two-year pilot program using direct-acting anti-viral HCV drugs (DAAS) to treat 8000 chronic patients in 2017 and another 19 500 in 2018 with 97% success rate, the NHI expanded DAAs coverage to all eligible patients at no cost in 2019 [25,26]. The Liver Disease Prevention and Treatment Research Foundation in Taiwan (Liver Foundation), a non-profit organization striving to eliminate liver diseases, began conducting nationwide community-based free blood and ultrasound screening programs for liver diseases in 1996 [27,28]. We utilized Taiwan as a case study to examine the relationship between hepatitis screening participation, awareness of infection status, and treatment-seeking behavior to learn valuable lessons for hepatitis prevention and eradication efforts around the world.

## METHODS

### Data source and participants

De-identified secondary data was obtained from the Liver Foundation’s community-outreach free blood-screening programs in 2015 (29 counties) and 2016 (45 counties) for a total of 56 197 participants covering a wide range of geographic locations and socioeconomic groups throughout the country. The screening activities were advertised through local TV, the radio, the Foundation website and social media, and community bulletin boards. All residents were encouraged to participate with no advanced registration required; the only criterion was that participants needed to be at least 20 years old, the legal age of adults in Taiwan. Individual consent was obtained prior to administering the survey and blood draw. Participants were incentivized with a small gift upon completion.

### Variables

The screening data had two parts: First, a questionnaire on basic demographics, screening history, self-reported hepatitis B and C infection status, treatment-seeking behaviors, and reasons for not following up with a medical professional for those previously aware they were or had been infected with hepatitis B or C (Table S1 in **Online Supplementary Document**). Second, blood test results which assessed HBsAg (indicative of active hepatitis B infection) and Anti-HCV (indicative of active or resolved hepatitis C infection). All participants who tested positive for HBsAg or Anti-HCV were asked to return for a free liver ultrasound screening and further follow-up instructions or referrals.

### Statistical methods

We removed 4637 participants that lacked either questionnaire or blood test results data, and another 651 for incomplete questionnaires, rendering 50 909 valid cases for analysis. Participants missing a single question were only removed from respective analyses case-wise.

Age was grouped into six categories from the 20s to 70 and older. Based on postal code, residential locations were divided into urban, mid-size/suburban, and rural categories, adopted from the classification of the 2009 Taiwan National Health Interview Survey [29]. R programming and chi-square tests were used to analyze the data [30].

### Ethics

The study was exempted from IRB because the analysis was completed utilizing a secondary, non-identifiable data set.

## RESULTS

This study population was 58.1% female with an average age of 52.0 years (standard deviation 15.3). By resident location, 26.3% of people lived in an urban area, 23.2% in a suburban area, and 50.5% in a rural area. Detailed participant demographics are available in **Table 1**.

**Table 1:** Participant demographics

Characteristic	Participants (n = 50 909)
**Gender:**	
Female	58.1%
Male	41.9%
Missing data	44
Age group (years)*	
20s	4.8%
30s	18.5%
40s	19.1%
50s	21.4%
60s	21.8%
70+	14.3%
Missing data	0
**Location:**	
Rural	50.5%
Average	23.2%
Urban	26.3%
Missing data	63

*Mean (standard deviation) age: 52.0 (15.3).

In terms of past screening rates and knowledge of their previous test results, 41.1% of participants indicated having had a hepatitis B or C screening before. About 16% of participants indicated having had a screening test but did not know the results. While 38.0% answered they never had a hepatitis screening, notably another 20.9% did not know whether they had or not. Knowledge level and screening history varied by demographic factors. Males were more likely to have had a screening but not know the results (18.2% male vs 14.7% female, *P* < 0.001), while females were more likely to not have had one (39.6% female vs 35.9% male, *P* < 0.001). Urban residents were more likely to have underwent a screening than those living in rural areas (*P* < 0.001). Despite low rates of prior screenings, 69.7% and 66.5% of participants self-reported awareness of their HBV and HCV infection status respectively (ie, self-reported they were or were not infected). Detailed screening history and awareness rates are available in **Table 2**.

**Table 2:** Screening history and self-reported awareness

	TOTAL	Gender		Age group (years)						Resident location		
*Total n = 50 909*	**Female**	**Male**	**20s**	**30s**	**40s**	**50s**	**60s**	**70+**	**Urban**	**Suburban**	**Rural**
**Self-reported they had been screened for HBV or HCV before**												
-Yes, and knew the result	25.0%	25.2%	24.7%	21.5%	26.9%	29.3%	25.2%	23.5%	19.9%***	26.7%	24.7%	24.2%***
-Yes, but did not know the result	16.1%	14.7%	18.2%***	13.5%	14.3%	17.4%	17.0%	17.5%	14.3%***	17.4%	16.9%	15.2%***
-No	38.0%	39.6%	35.9%***	39.7%	36.4%	34.2%	39.0%	39.8%	40.7%***	35.2%	37.7%	39.7%***
-Did not know	20.9%	20.5%	21.3%*	25.5%	22.4%	19.1%	19.0%	19.3%	25.2%***	20.7%	20.8%	21.0%
Missing data	672	713		672						733		
**Self-reported awareness of if they were or were not infected with HBV**	69.7%	70.5%	68.5%	65.6%	73.8%	73.6%	71.3%	68.0%	60.6%	72.7%	69.4%	68.2%
Missing data	176	220		176						239		
**Self-reported awareness of if they were or were not infected with HCV**	66.5%	67.7%	64.8%	64.9%	70.5%	68.5%	67.9%	65.2%	59.3%	69.0%	66.0%	65.5%
Missing data	502	540		502						562		

HBV – hepatitis B virus, HCV – hepatitis C virus

Asterisks indicate χ^2^ significance:*P < 0.05, **P < 0.01, ***P < 0.001.

For hepatitis B, 12.3% of participants tested positive for HBsAg, indicating they had an active acute or chronic HBV infection. More males tested positive than females (14.3% males vs 10.9% females, *P* < 0.001). The prevalence rates peaked for participants in their 40s (17.0%). Of those who tested positive for HBV, 65.5% were correctly aware of their infection status (ie, self-reported they were infected) while 12.8% were incorrectly aware of their infection status (ie, self-reported they were not infected); females, older participants, and rural residents were significantly more likely to have incorrect awareness. Of those who tested negative for HBV, few people (2.81%) were incorrectly aware of their infection status (ie, self-reported they were infected). Detailed results for hepatitis B are available in **Table 3**.

**Table 3:** Hepatitis B Blood test results and correct awareness

	Total	Gender		Age group (years)						Resident location		
*Total n = 50 909*	**Female**	**Male**	**20s**	**30s**	**40s**	**50s**	**60s**	**70+**	**Urban**	**Suburban**	**Rural**
**HBsAg blood test results:**												
POSITIVE (n = 6249)	12.3%	10.9%	14.3%***	2.54%	12.5%	17.0%	13.8%	11.5%	8.02%***	11.6%	13.1%	12.2%**
NEGATIVE (n = 44 660)	87.7%	89.1%	85.8%***	97.5%	87.5%	83%	86.2%	88.5%	92.0%***	88.4%	86.9%	87.8%
Missing data	0	44		0						63		
**Of those POSITIVE, participants who:**												
Correctly self-reported infected	65.5%	62.7%	68.4%***	56.5%	73.3%	70.3%	65.6%	62.2%	44.0% ***	68.8%	71.0%	61.1%***
Incorrectly self-reported not infected	12.8%	14.5%	10.9%***	19.4%	8.34%	9.98%	12.4%	13.9%	27.0%***	11.3%	8.97%	15.3%***
Did not know	21.8%	22.8%	20.8%	24.2%	18.4%	19.7%	22.0%	23.9%	29.0%***	19.9%	20.1%	23.6%**
Missing data	36	48		36						46		
**Of those NEGATIVE, participants who:**												
Correctly self-reported not infected	66.0%	68.0%	63.1%***	64.6%	71.6%	70.0%	67.3%	63.3%	56.7%***	69.2%	65.2%	64.6%***
Incorrectly self-reported infected	2.81%	2.05%	3.90%	0.76%	1.20%	2.40%	3.35%	4.10%	3.38%	2.74%	2.97%	2.78%
Did not know	31.2%	30.0%	33.0%	34.7%	27.2%	27.6%	29.4%	32.7%	39.9%	28.0%	31.8%	32.6%
Missing data	140	172		140						193		

HBV – hepatitis B virus

Asterisks indicate χ^2^ significance: *P < 0.05, **P < 0.01, ***P < 0.001.

For hepatitis C, 3.53% of participants tested positive for Anti-HCV, indicating they had an active acute, chronic, or resolved HCV infection. Those who were older and those living in a more rural location were more likely to be or have been infected with HCV (5.05% rural vs 1.64% urban, *P* < 0.001). Of those who tested positive for Anti-HCV, 44.6% were correctly aware of their infection status (ie, self-reported they were infected) and 26.4% were incorrectly aware of their infection status (ie, self-reported they were not infected). Gender, age, and location were not significant indicators of incorrect awareness, while participants in their 40s to 60s generally had more correct awareness. Of those who tested negative for Anti-HCV, few (0.72%) were incorrectly aware of their infection status (ie, self-reported they were infected). Detailed results for hepatitis C are available in **Table 4**. One hundred and ninety-four participants (0.381%) tested positive for both HBsAg and Anti-HCV.

**Table 4:** Hepatitis C blood test results and correct awareness

	Total	Gender		Age group (years)						Resident location		
Total n = 50 909	**Female**	**Male**	**20s**	**30s**	**40s**	**50s**	**60s**	**70+**	**Urban**	**Suburban**	**Rural**
**Anti-HCV blood test results:**												
POSITIVE (n = 1795)	3.53%	3.50%	3.56%	.370%	1.04%	1.88%	3.30%	5.01%	8.05%***	1.64%	2.36%	5.05%***
NEGATIVE (n = 49 114)	96.5%	96.5%	96.4%	99.6%	99.0%	98.1%	96.7%	95.0%	91.9%***	98.4%	97.6%	95.0%***
Missing data	0	44		0						63		
**Of those POSITIVE, participants who:**												
Correctly self-reported infected	44.6%	42.4%	47.7%*	22.2%	40.2%	49.7%	49.9%	48.7%	37.0%***	43.1%	50.4%	43.9%
Incorrectly self-reported not infected	26.4%	27.6%	25.0%	44.4%	27.8%	28.4%	25.5%	24.2%	28.0%	25.2%	24.3%	26.4%
Did not know	28.9%	30.1%	27.4%	33.3%	32.0%	21.9%	24.7%	27.1%	35.0%**	31.7%	25.4%	29.6%
Missing data	10	10		10						10		
**Of those NEGATIVE, participants who:**												
Correctly self-reported not infected	66.3%	67.6%	64.5%***	64.9%	70.3%	64.0%	67.7%	64.6%	58.1%***	69.3%	65.8%	65.0%***
Incorrectly self-reported infected	0.72%	0.59%	0.88%	0.25%	0.34%	0.42%	0.86%	1.01%	1.09%	0.43%	0.60%	0.93%
Did not know	33.0%	31.8%	34.6%	34.9%	29.3%	35.6%	31.4%	34.4%	40.8%	30.3%	33.6%	34.1%
Missing data	492	530		492						552		

HCV – hepatitis C virus

Asterisks indicate χ^2^ significance: *P < 0.05, **P < 0.01, ***P < 0.001.

Pertaining to subsequent action, of those who self-reported they were infected with HBV or HCV, only 56.6% and 73.4% respectively indicated they had been following up for monitoring or treatment. Males (60.1% male vs 52.8% female, *P* < 0.001) and older age groups were more likely to monitor their hepatitis B; resident location showed no impact. The demographic differences were not significant for hepatitis C.

Among those who self-reported they were infected with HBV or HCV and were not following up, the most common reasons for not doing so were “no symptoms or discomfort” (62.9%, *P* < 0.001 difference by location with urban and suburban more likely to list this answer choice as one of the reasons), followed by “too busy and no time” (37.0%, *P* < 0.001 difference by age with younger groups more likely to list), “I do not know where to follow-up” (10.9%, *P* < 0.001 difference by location with more urban areas more likely to list), “inconvenient to take a day off” (9.49%, *P* < 0.001 difference by age with younger groups more likely to list), and “trying to avoid medical expenses” (2.47%; *P* < 0.05 difference by age and *P* < 0.01 difference by location with younger and more urban participants more likely to list). Note that participants could list more than one answer. Detailed results on follow-up are available in **Table 5**.

**Table 5:** Follow-up rates and reasons for not following up

	Total	Gender		Age group						Resident location		
**Female**	**Male**	**20s**	**30s**	**40s**	**50s**	**60s**	**70+**	**Urban**	**Suburban**	**Rural**
**Of those who self-reported awareness they were infected with HBV (n = 5325), participants who have followed up**	56.6%	52.8%	60.1%***	53.9%	50.5%	56.4%	56.3%	59.7%	65%***	55.5%	55.3%	58.1%
Missing data	944	984		944						948		
**Of those who self-reported awareness they were infected with HCV (n = 1140), participants who have followed up**	73.4%	73.5%	73.2%	40%	71.1%	70.5%	69.3%	75.9%	76.8%	68.9%	71.5%	75%
Missing data	351	352		351						351		
**Of those who self-reported awareness they were infected and not following up (n = 2110), reasons for not following up:†**												
-No symptoms or discomfort	62.9%	63.7%	62.1%	73.3%	59.9%	64.2%	61.8%	61.4%	76.1%	66.8%	68.6%	56.6%***
-Too busy or no time	37.0%	35.4%	38.7%	53.3%	43.3%	43.9%	33.8%	26.7%	27.2%***	38.8%	33.0%	38.6%
-Don’t know where to go for follow-up	10.9%	10.1%	11.7%	0%	13.5%	10.7%	10.2%	11.2%	5.43%	15.1%	12.1%	7.47%***
-Inconvenient to take day off	9.49%	8.52%	10.5%	10.2%	13.3%	16.3%	12.7%	6.12%	3.95%***	8.98%	8.88%	10.3%
-Trying to avoid medical expenses	2.47%	2.75%	2.18%	6.67%	3.44%	4.06%	1.46%	0.99%	0%*	4.37%	2.28%	1.40%**
Missing data	615	615		615						617		

HBV – hepatitis B virus, HCV – hepatitis C virus

Asterisks indicate χ^2^ significance: *P < 0.05, **P < 0.01, ***P < 0.001.

†n = 372 (24.9%) participants chose two or more reasons.

## DISCUSSION

During the first World Hepatitis Summit in 2015, Glasgow Declaration on Hepatitis committed all governments to eliminate viral hepatitis [17]. Successful policies and programs in prevention and treatment require the public’s awareness, attention, and action [31]. This study revealed several disconnections in the recognition and behavior steps necessary for hepatitis eradication and liver disease prevention, some of which are critical yet often overlooked in extant literature and policy discussion.

It is surprising that 37.0% of the participants either did not know whether they had undergone a hepatitis test or did not know the results of a previous screening. This could be due to several factors. Hepatitis screening may be done in conjunction with other blood screening, so participants are uncertain if they have been tested. Others may not understand the laboratory reports, get confused with different diseases, or not pay attention when the blood test results are normal or negative. Moreover, our findings showed that 69.7% and 66.5% of the participants self-reported awareness of their HBV and HCV infection status respectively. Though the percentages are not optimal, they are higher than most others across the world; previous studies have reported hepatitis awareness rates in the United States and European Union ranging from 8.8% to 80%, less than 10% in South-East Asia, and less than 5% in Africa [12,13,15,16,32]. A 2019 publication on national awareness of viral hepatitis indicated that 33.9% and 55.6% of the Americans participants were aware of their chronic HBV and HCV infection respectively [33]. These statistics all point to a clear need for more blood screening programs together with more explicit communication to better inform the public of their hepatitis status and actionable prevention measures.

Self-reported awareness rates not verified against blood test results may be misleading and overestimate the public’s knowledge of their hepatitis status. Though only 41.1% of participants indicated a prior screening, about two thirds of participants reported self-awareness of their hepatitis B and C status. Some participants may have believed they knew their infection status despite no screening history or not remembering a blood test because of a lack of symptoms or high-risk behaviors. Our study further verified the self-reported awareness. Among the participants who tested positive for HBsAg and Anti-HCV, 12.8% and 26.4% respectively were incorrectly aware of their infection status (ie, self-reported they were not infected). Combined with those who were unaware of this infection status, 34.6% and 55.3% of the HBV and HCV infected population are at jeopardy of not discovering their hepatitis. This could result in a lack of proper treatment until later stage liver disease or cancer as well as more unrestrained involvement in higher risk behaviors that intensify damage to the liver (ie, excessive alcohol) or increase the likelihood of transmitting HBV or HCV to others (ie, injection drug use).

Even with self-reported awareness of an infection, only 56.6% and 73.4% in our study were monitoring their condition or seeking treatment with a medical professional for HBV and HCV respectively. Similarly, less than 60% of HCV-infected people in New York City and 77.5% of HCV-infected people in the United States follow up [12,32]. Such deficiency in patient action defeats screening efforts and halts the progress of eliminating hepatitis and preventing liver diseases at the individual and global levels [11]. While self-reported awareness rates of infection status was fairly similar for HBV and HCV, the findings showed more people were monitoring their HCV than those with HBV. It is possible that the recent publicity both in Taiwan and the United States surrounding improved efficacy of the new direct-acting hepatitis C drugs has generated more attention to HCV.

In terms of health disparity between geographic locations, our study found evidence of inequality: those from rural areas had lower rates of screening participation as well as both awareness and correct awareness of their infection status. This shows the need for more prevention, education, and treatment programs in rural regions. Unexpectedly, participants in rural areas were least likely to list “no pain or symptoms”, “don’t know where to go for follow-up”, or “trying to avoid medical expenses” as reasons to not follow-up after being diagnosed. This could speak to the successful health care delivery system in place in Taiwan that specifically works to increase health education and access to health resources in disadvantaged areas. On the other hand, there is also a need to better educate both urban and more rural-located residents on the seriousness of hepatitis and treatment options available. It is important to note, however, that the order of most frequently listed reasons for not following up after being diagnosed by rural participants mirrored that of urban and suburban participants. Further, there were significant differences in the rates of selecting “too busy or no time” and “inconvenient to take day off” among age groups but not across gender or resident locations. Younger people more frequently reported lack of time or inconvenience in taking a day off, likely due to their working schedule. Remarkably, only 2.47% of participants picked medical cost as the reason for not following up. This could be testament that Taiwan’s health insurance, which includes substantial coverage for hepatitis and other treatments, has effectively alleviated the public’s financial burden. Taiwan’s universal health system is well-established throughout the country and is largely accessible and affordable. In rural areas, health services are generally provided at local health centers or through integrated delivery system (eg, regular mobile clinics) [34]. In addition to the free screening offered by the Liver Foundation, Taiwanese citizens can request hepatitis testing at clinics or hospitals for approximately US$5 to US$20 out-of-pocket, which is waived for low-income and aboriginal residents [35]. For high-risk populations or suspected cases, providers may order tests at no or low co-pays for patients. Positive individuals can register for free monitoring and treatment programs that include abdominal sonographies, liver function tests every six months, and medication coverage for up to 36 months [36].

There are some limitations to this study. Participants were self-selected to participate in the screening which introduced inherent bias to the results. Though the screening program had no eligibility restriction other than being at the age of 20 or above, the sample had disproportionally larger percentages of seniors and people living in rural areas. This is likely owing to the Liver Foundation’s priority and stronger promotion of screening among underserved population and the fact that almost everyone under age 30 in Taiwan receives HBV vaccines at birth. The results may overestimate the rate of hepatitis infection relative to the general population of Taiwan. Though the specific statistics reported in this study may not be generalizable to all countries, the findings highlight noteworthy patterns of disparity and areas of common concerns in global hepatitis prevention and reduction. Further, the survey was designed and implemented by the Liver Foundation staff prior to this study; we were unable to validate survey questions. The small number of questions in the survey precludes full understanding of why hepatitis awareness or treatment behaviors vary by demographic factors. Future research could use qualitative methods to explore the factors attributed to incorrect awareness and the disconnect in awareness and actions to help elucidate these variations and discrepancies. As previously mentioned, Anti-HCV is positive for those with resolved HCV infections. These results did not capture participants who tested positive because of a resolved infection and were thus correctly aware of their infection status (ie, self-reported they were not infected) or incorrectly aware of their infection status (ie, self-reported they were infected). A small portion of the participants who incorrectly self-reported they were not infected with HBV or HCV could have been infected since their last screening. In addition, missing data could lead to some misrepresentation of results. A number of participants who self-reported awareness that they were infected with HBV or HCV did not respond to whether they have followed up, perhaps due to denial of the disease, unawareness of treatment options, or discomfort admitting that they did not follow-up.

## CONCLUSIONS

Our study revealed that the percentages of people uncertain of their screening history, unaware of their test results, and with incorrect awareness of their hepatitis status are significant, even in countries that provide universal, high-quality health care like Taiwan. Health prevention and intervention programs worldwide need to reconsider their approaches to raising awareness and ensuring correct awareness of hepatitis infection and proper follow-up with a health professional. Future screening efforts should strive to clearly notify people of hepatitis testing events and explain testing results, both positive and negative. They should also more strongly urge people who tested positive to follow up with health care providers for treatment and/or continued monitoring to prevent the development of life-threatening issues and costly medical expenditure for the individuals and the system later on. The expansion and adoption of electronic medical records could allow more direct access for the public to review their test results and help achieve the intended purpose of screening by improving awareness of hepatitis as well as other disease status.

Rural areas had lower rates of blood testing for hepatitis and thus require more screening program efforts. In contrast, in more urban areas there is a striking need for more focused health education to inform the public about the seriousness and spreadability of hepatitis despite the absence of symptoms as well as to better publicize locations people may go to receive hepatitis care. Health education should also convey that there now are effective medications for both hepatitis B and C in order to encourage the public, and especially those who tested positive, to come back periodically for regular hepatitis monitoring. It is important to note that while perceptual access to follow-up care requires improvement, those tangible resources must be readily available to fulfill those needs. Urban and younger populations often complain of no time or inconvenience with work; employee compensation could be considered in order to convince more of this demographic to follow-up with a positive test result.

Wide-scale blood screenings are an imperative tool necessary to help reach the Hepatitis Summit’s goal of eradication and global efforts to reduce the burden of liver diseases such as cirrhosis and cancer. Our study results provide insights on the implementation and efficacy of such a crucial program and recommendations for future improvements.

## Additional Material

Online Supplementary Document
