# Supplementary material for: The disconnect in hepatitis screening: participation rates, awareness of infection status, and treatment-seeking behavior
Source: J Glob Health. 2019 Apr 24;9(1):010426. doi: 10.7189/jogh.09.010426 (PMC6486119; doi:10.7189/jogh.09.010426)
Supplement: Online Supplementary Document [file jogh-09-010426-s001.pdf]

J Glob Health 2019; 9: 010426

Online Supplementary Document

Table S1. Brief Hepatitis Screening Questionnaire

	Question	Answer choices		
1)	Do you have hepatitis B?	a. Yes	b. No	c. Don't know
2)	Do you have hepatitis C?	a. Yes	b. No	c. Don't know
3)	Have you been screened for HBV or HCV before?	a. Yes, but don't know the result<br>b. Yes, know the result<br>c. No<br>d. Don't know		
4)	(only for people self-reported being infected with HBV)<br>Since you became aware of your infection, have you been following up with a healthcare provider?	a. Yes	b. No	
5)	(only for people self-reported being infected with HCV)<br>Since you became aware of your infection, have you been following up with a healthcare provider?	a. Yes	b. No	
6)	(only for people self-reported being infected with HBV or HCV)<br>What are the reasons that you have not been following up or seeking treatment after becoming aware of your infection? Check all that applied.	a. no symptom or discomfort<br>b. too busy, no time<br>c. inconvenient to take off from work<br>d. transportation issue<br>e. to avoid extra medical expenses<br>f. don't know where to follow up or get treatment<br>g. no treatment facility in the proximity<br>h. have used alternative measure or supplements instead<br>i. other:		
